# Do Migrant Residents Really Achieve Health Equity by Obtaining Urban Hukou? A Comparative Study on Health Service Utilization and Urbanization in Central China

**DOI:** 10.3389/fpubh.2022.784066

**Published:** 2022-04-05

**Authors:** Rui Min, Zi Fang, Chunyan Zi, Changmin Tang, Pengqian Fang

**Affiliations:** ^1^School of Public Health, Tongji Medical College, Huazhong University of Science and Technology, Wuhan, China; ^2^Tongji Hospital, Tongji Medical College, Huazhong University of Science and Technology, Wuhan, China; ^3^Wuchang Hospital, Wuhan, China; ^4^School of Management, Hubei University of Chinese Medicine, Wuhan, China; ^5^Tongji Medical College, Huazhong University of Science and Technology, Wuhan, China; ^6^Academy of Health Policy and Management, Huazhong University of Science and Technology, Wuhan, China

**Keywords:** urbanization progress, rural-to-urban residents, health service utilization, equity in health, comparative study

## Abstract

**Introduction:**

With more than 120 million rural-to-urban migrants, urbanization of the rural population requires deeply exploration in China.

**Objective:**

This study focused on settled citizens who obtained urban Hukou (household registration) during urbanization and investigated their perceptions of health services in China.

**Method:**

A cross-sectional comparison study with an original, closed questionnaire was conducted in two major cities of Hubei, central China, covering health status and both the satisfaction with and utilization of health services. In total, 863 residents with urban Hukou participated in this study; migrants formed the study group and original city residents formed the control group. Propensity score matching (PSM) was used to reduce choice bias in the analysis steps. Besides basic description of the data, ordinary least squares regression (OLS regression) was used to discover the relationship between basic demographic indicators and health expenditure.

**Results:**

PSM yielded 290 effective pairs for analysis. The results indicated an improvement in health status for migrant residents (study group) with a higher average score of self-reported health status and lower prevalence of chronic diseases than the control group. These scores were also better than the standard urban level in central China. The study group showed a higher clinic visit utility (69.63%), lower hospitalization utility (8.28%), less convenience of health service utility, and lower health expenditure than the control group. For the study group, the biggest difference was observed in satisfaction with health service costs, which was the least improved aspect after they obtained urban Hukou. The regression results demonstrated that age, family size, living expenditures, and marital status impacted health costs in the overall model and the influences of these factors differed between the study and control groups.

**Conclusions:**

Obtaining urban Hukou helps migrant residents to meet their health service needs and receive equal access to health services. However, after obtaining urban Hukou, migrants also face great pressure in terms of health consumption. This study therefore offers guidance on the next steps for progressing China's urbanization.

## Introduction

Urbanization is a natural historical process following the development of industrialization, which includes the urban concentration of non-agricultural industries and the migration of rural populations to urban areas (1, 2). China was similar to other countries in the initial stages of industrialization, with rural-urban migration accounting for a large proportion of urbanization growth (2–5). Along with the acceleration of industrialization, China's urbanization experienced a low starting level and fast development. As a country with the largest global population, China has experienced unprecedented human migration from rural to urban areas (6). By the end of 2020, China had 902 million urban residents, more than triple the number 40 years prior, during the Reform and Opening of China in 1978; this number is still rising as urbanization accelerates (7). An additional 430 million rural-to-urban migrants are anticipated over the next 25 years, with China targeting an urbanization rate of over 80%.

Rapid urbanization creates significant changes and influences social development and lifestyles (6, 8). It provides opportunities to improve life quality after rural-to-urban migration and significantly impacts public resources, such as health, education, occupations, income, and life (1, 5). With the shifting and aggregating population, urbanization in China has challenged the equity and safety of the social and natural environments and placed pressure on public health (9, 10), particularly on the prevention and control of infectious and chronic diseases. In addition, urbanization has limited the quality of health resources and has increased health expenditure and disease burdens (8, 11).

The urbanization of the rural population, or becoming an urban resident (referred to as new city citizens in this research) in China is an issue requiring exploration (12). The disparities between rural and urban areas include, not only economic, but also social factors, such as growing environments, education backgrounds, traditional customs, as well as the benefits and priority provided by the unique household registration system (Hukou). It has been reported that Hukou, which is a product of dual governance in urban and rural areas, shows great power in granting equity of health service supply (13). Authorities and researchers have explored the utility of health services and the development of healthcare systems, focusing on migrants without urban Hukou (known as floating people); further they have discussed the unfairness and problems in health utility and supply (10, 11, 14).

In the face of contradictions and challenges during urbanization and new requirements for improving the overall quality of urbanization, the Central Committee of the Communist Party of China and the State Council have issued a series of relevant policy documents, focusing on solving the existing rural-to-urban migration problems (12). A three-step experimental reform was launched in 2014, with the first of 62 pilot areas focused on migrant groups whose lives have changed significantly in the process of urbanization, particularly safeguarding their basic rights as new city citizens (15). Following the establishment of this policy, urbanization in China accelerated, with over 120 million migrants settling as new city citizens. For rural-to-urban migrants, better integration into city life and changing lifestyles and behaviors are more important than the shift in living place during the urbanization progress (6). However, their voices are hardly heard. Unlike the floating population, whose health rights have been widely discussed, the basic rights of migrant groups settling in the city, undergoing a civil transformation, have not been well-explored. The following questions are yet to be answered: What happens to rural-to-urban migrants after obtaining urban Hukou? Does the policy effectively promote social equity? What should be the next steps? There is an urgent need to know the answers to these questions, for both the public and the relevant authorities.

Therefore, this study aimed to prove the effectiveness of China's new urbanization reform, and provide evidence for the trend of urbanization by examining health equity in the process of urbanization. It also attempted to understand the social situation of rural-to-urban migrants and the changes they face after obtaining urban Hukou.

## Materials and Methods

### Study Design and Setting

Face-to-face survey with closed questionnaires were conducted to investigate the equity of health service delivery in the process of urbanization and to identify the disparities and influencing factors of health service demands. The aim was to gain insights that could facilitate effective and precise policy development.

This cross-sectional comparison survey was conducted *via* random sampling (see Section Participants and Procedure) from January to June 2018 in Wuhan and Xiangyang, two cities in Hubei, which were the top 10 cities for urbanization and economic development in central China (see Appendix 1).

The study was approved by the Ethics Committee of Tongji Medical College, Huazhong University of Science and Technology (IORG No: IORG0003571). Personal identifying information was removed and participants remained anonymous during the entire study process. The research team kept all paper documents and electronic data secure.

### Participants and Procedure

A unified definition of the new city citizen group is lacking, as they emerged less than a decade ago. To clarify the research objective, this study defined new city citizens as people who have moved from rural areas to work and settle in cities. To understand how the new city citizen group changed throughout the urbanization process, a quasi-experimental research strategy was adopted in this study. Specifically, a study group and control group were created as part of the cross-sectional survey. A specific section in the questionnaire for the study group was designed to determine the health service satisfaction before and after settle-down.

### Design of Study Group and Control Group

For the study and control groups, we selected migrant residents and original residents, respectively; both groups had urban Hukou, to avoid any confounding effect of the policy acting as an extraneous variable.

In each surveyed city, three districts/county-level cities with comparable levels of economic development were selected. A total of 900 people (sampling ratio, 2:1, *study group vs. control group*) from communities in the sample area were selected through random sampling (see Appendix 2).

The inclusion criteria were citizens over 18 years of age with the ability to think and answer independently. Individuals who were from rural areas before receiving urban Hukou, with a settlement period of <10 years, formed the study group. City residents who had urban Hukou from birth, called as original residents in this study, formed the control group.

The exclusion criteria were: (1) college students with a school collective Hukou, and (2) those with no change in Hukou status after settling down (termed floating people). Based on previous research experience and a literature review, questionnaires with over 20% missing data were excluded (16, 17).

### Design to Reflect Changes in the Urbanization Process

The identity of migrant citizens shifts from farmer, to floating people, to new city citizen. As the study focused on settled citizens who had obtained urban Hukou during the urbanization process, we asked the study group about their experience regarding the use of health services before and after they had obtained urban Hukou, to identify changes in health services.

The questionnaire was distributed utilizing the convenience sampling method in the sample communities by professional investigators. All investigators received relevant training prior to the investigation. Both written and oral informed consent was obtained from all participants before conducting the survey. After obtaining permission, the respondents were asked to complete a paper-based questionnaire in a face-to-face setting.

### Survey Content and Indicators

This study used an original questionnaire with four sections as shown in Appendix 3. The questionnaire included mainly closed-ended questions. Section Introduction was the demographic information. Sections Materials and Methods and Discussion were designed to be consistent with the National Health Service survey (18). And Section Results was the satisfaction with health service combined with the goal of China's health service system reform. Details regarding study indicator types and value assignment are presented in Appendix 4.

The content validity index (CVI) for the questionnaire was 0.704, whereas item CVI ranged from 0.67 to 0.73. The results of the validity test of the questionnaire's reliability (Cronbach's α = 0.70, KMO = 0.70) indicated good internal consistency. The correlation coefficient test (*P* <0.01) demonstrated good structural validity among the dimensions and the overall score. The different dimensions are elaborated below:

### Demographic Information

A total of nine items involving details such as gender, age, educational background, marital status, registration information, occupation status, family members, and household economic status (annual family income and living expenditure) were obtained.

### Health Status

Self-reported health status, chronic disease history, and self-reported illnesses over the previous two weeks were used to evaluate health status.

### Satisfaction With Health Services

The central authority of China launched the new round reform of health service system in 2009, aimed to establish a safe, effective, convenient and affordable health service system. Referring to the goal of the new round reform of health service system, this study gave a four questions satisfaction evaluation with health services, including technology level, expenditure, convenience, service attitude. And the study group was asked to answer a separated part, “changes of satisfaction with health services before and after settling down”. The two-part section used a 6-point Likert scale (0 = I don't use it, 1 = very bad/much worse, 2 = bad/worse, 3 = neutral, 4 = good/better, 5 = very good/much better).

### Healthcare Service Utilization

Affordability, access, health needs, and behaviors were evaluated. Affordability included health insurance coverage and health expenditure. Access included convenience in terms of distance and time taken to reach the nearest health institution. Healthcare service usage included use of clinical and in-hospital services.

### Data Collection and Statistical Analysis

Data were obtained from questionnaires and official statistical reports. EpiData 3.0 (The EpiData Association, Odense, Denmark) was used for data entry, and SPSS26.0 (IBM, USA) was used for statistical analysis with coefficients equality estimation replenished by SPSSAU (version 21.0, Online Application Software). Propensity score matching (PSM) was used to reduce the choice bias in the analysis steps. With one-to-one matches without replacement, more balanced groups were matched with a set of baseline characteristics, including investigated city, sex, age, marital status, education background, family income, occupational status, and family size (see Appendix 5).

Univariate and bivariate statistical models were adopted to evaluate the data. Continuous variables were described using mean and standard deviation (SD), while non-normally distributed data were described using median and quartile range (25^th^ and 75^th^ percentiles). Categorical variables were reported as counts and percentages. The normally distributed continuous data were tested using *t*-test, non-normally distributed using the Kolmogorov-Smirnov test, and categorical data using the chi-square test. After the correlation analysis, related basic demographic indicators and health service indicators were incorporated into the regression model (ordinary least squares regression, OLS) for the multiple analysis of health service utility (dependent variable = health expenditure). Then, the non-normality test, the related continuous data was transformed for the regression model improvement. Furthermore, this study attempted to determine whether the independent variables had the same influence on the dependent variable in different citizen groups. Regression analysis was used for both the study group and control group. The equality of regression coefficient for the two groups was tested (*t*-test and Chow test) to compare the difference of influence between the two groups. Statistical significance was set at *P* < 0.05 for a two-sided test.

## Results

A total of 819 participants were included in the study, with an effective response rate of 94.9%. There were 529 (64.6%) participants in the study group and 290 participants in the control group. After the PSM analysis, 290 effective pairs and 580 cases were obtained for research analysis at the ratio level of 1:1.

### Demographic Information and Health Status

The average age of all 580 participants was 38.95 (±11.35, 20, 83). Of all the participants, 53.79% (*n* = 312) were women, 75.34% were married, and 88.28% were employed. The median family size was three people, and families comprising three or four was common (84.3%). Among the study group, the sex ratio was 1:1.25 (men to women), the average age was 39.99 years (±11.22), and 72.41% were married (Table 1).

**Table 1:** Demography characteristics of migrant group compared to aboriginal group before and after propensity score matching (PSM).

**Characteristics**	**Before PSM**			**After PSM**		
	**Control group Original *N* = 290**	**Study group Migrant *N* = 529**	***p*-value**	**Control group Original *N* = 290**	**Study group Migrant *N* = 290**	***p*-value**
Age (mean ± S.D.)	39.36 ± 11.77	35.94 ± 10.17	<0.001	39.36 ± 11.77	38.55 ± 10.92	0.392
BMI (mean ± S.D.)	22.08 ± 3.24	22.18 ± 2.96	0.649	22.08 ± 3.24	22.48 ± 3.12	0.131
Self-report Health status (mean ± S.D.)	70.40 ± 16.40	73.97 ± 15.15	0.002	70.40 ± 16.40	74.62 ± 14.70	0.003
Family income [median, (p25, p75)]	100,000 (6,000, 126,399)	100,000 (58,000, 150,000)	0.083	100,000 (6,000, 126,399)	100,000 (50,000, 150,000)	0.495
Living expenditure [median, (p25, p75)]	60,000 (35,750, 93,603)	50,000 (30,000, 93,603)	0.014	60,000 (35,750, 93,603)	50,000 (30,000, 93,603)	0.013
Health expenditure [median, (p25, p75)]	10,000 (4,250, 20,000)	6,000 (2,500, 10,000)	0.058	10,000 (4,250, 20,000)	5,000 (2,000, 10,000)	0.011
**Gender**, ***n*** **(%)**						
Male	139 (47.93%)	236 (44.61%)	0.362	139 (47.93%)	129 (44.48%)	0.405
Female	151 (52.07%)	293 (55.39%)		151 (52.07%)	161 (55.52%)	
**Marital status**, ***n*** **(%)**						
Married	212 (73.10%)	376 (71.08%)	0.538	212 (73.10%)	210 (72.41%)	0.852
Single/widow/divorce	78 (26.90%)	153 (28.92%)		78 (26.90%)	80 (27.59%)	
**Education background**, ***n*** **(%)**						
Middle school and below	51 (17.59%)	98 (18.53%)	0.004	51 (17.59%)	71 (24.48%)	0.020
High school or technical college	154 (53.10%)	200 (37.81%)		154 (53.10%)	122 (42.07%)	
Bachelor and above	85 (29.31%)	231 (43.67%)		85 (29.31%)	97 (33.45%)	
**Occupation status**						
Employed	257 (88.62%)	465 (87.90%)	0.667	257 (88.62%)	255 (87.93%)	0.600
Retired	19 (6.55%)	23 (4.35%)		19 (6.55%)	16 (5.52%)	
Unemployed or else	14 (4.83%)	41 (7.75%)		14 (4.83%)	19 (6.55%)	
**Family members**, ***n*** **(%)**						
1–2 people	17 (5.86%)	48 (9.07%)	0.151	17 (5.86%)	19 (6.55%)	0.591
3–4 people	242 (83.45%)	431 (81.47%)		242 (83.45%)	247 (85.17%)	
5 people-	31 (10.69%)	50 (9.45%)		31 (10.69%)	24 (8.28%)	
**Chorionic diseases history**, ***n*** **(%)**						
Yes	71 (24.48%)	97 (18.34%)	0.008	71 (24.48%)	63 (21.72%)	0.225
No	184 (63.45%)	390 (73.72%)		184 (63.45%)	202 (69.66%)	
I'm not sure	35 (12.07%)	42 (7.94%)		35 (12.07%)	25 (8.62%)	
**Self-reported illnesses over the previous 2 weeks**						
Yes	100 (34.48%)	280 (52.93%)	<0.001	190 (65.52)	135 (46.55)	<0.001
No	190 (65.52%)	249 (47.07%)		100 (34.48)	155 (53.45)	
**Self-medication behavior**						
Yes	77 (26.55%)	170 (32.14%)	0.096	213 (73.45)	219 (75.52)	0.851
No	213 (73.45%)	359 (67.66%)		77 (26.55)	71 (24.48)	

*PSM, Propensity Score Matching*.

No statistically significant differences were found between the study and control groups in terms of gender, age, and marital status; however, the control group had a higher education level (*p* <0.01), occupation rate (*p* = 0.04), and annual family expenditure [60,000 vs. 50, 000 Chinese yuan (RMB, *1 U.S. dollar* = *6.8985 RMB in 2019*); Table 1].

The average self-reported health status was 74.62 for the study group, which was 4.22 higher than the control group. The prevalence of self-reported illnesses over the previous 2 weeks in the study group was 17.59% lower than that of the control group, whereas self-medication behavior was common in both groups (73.10%, *n* = 424; Table 1).

### Satisfaction With Health Services

**Figure 1:**
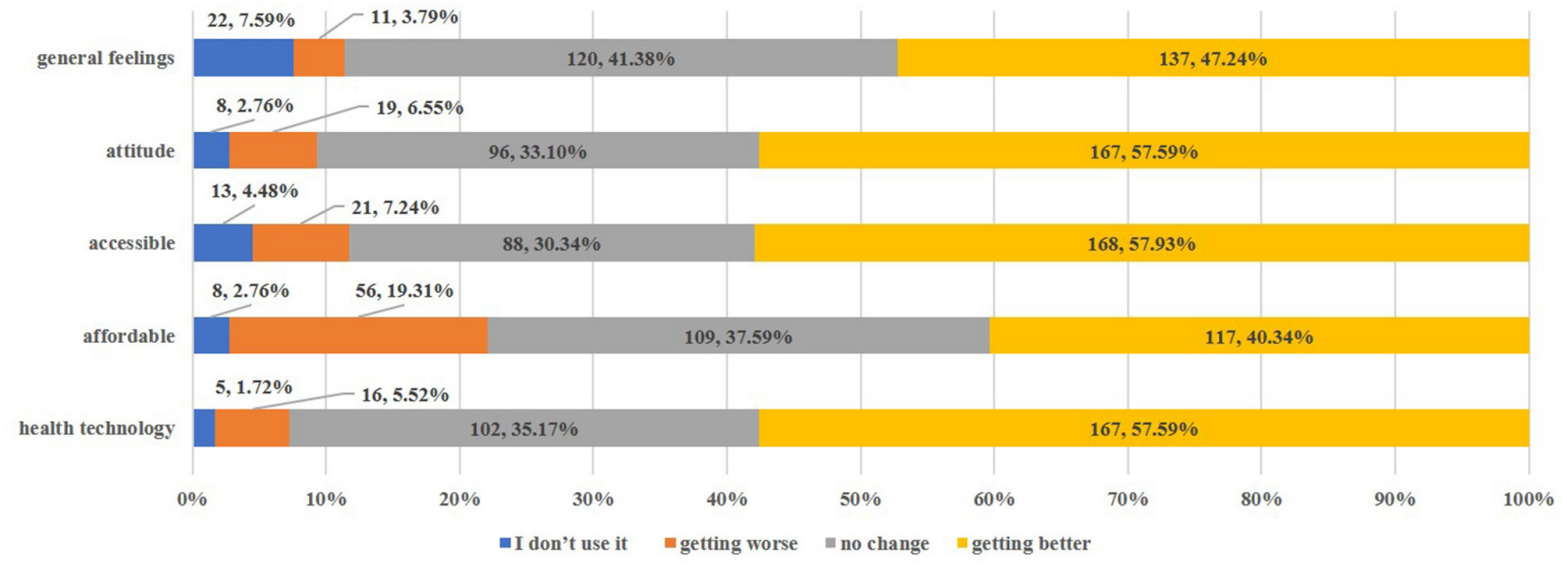
Change in health service utilization after obtaining urban Hukou (only migrants) [*n*, %].

Generally, over half of all the participants (52.59%) were satisfied with the health services, with no statistically significant differences between the two groups (Table 2). Meanwhile, 47.24% participants in the study group reported improvements in healthcare services compared with their prior location (Figure 1).

**Table 2:** Satisfactions evaluation for health service among all interviewees and two groups.

**Items**	**Type of Citizen (%)**		**Total (%) *N* = 580**	**χ^2^**	***p***
	**Control group *N* = 290**	**Study group *N* = 290**			
**General feelings for health service**					
I don't use it	18 (6.21)	18 (6.21)	36 (6.21)	5.128	0.400
Very bad	9 (3.10)	3 (1.03)	12 (2.07)		
Bad	18 (6.21)	15 (5.17)	33 (5.39)		
Neutral	102 (35.17)	92 (31.72)	194 (33.45)		
Good	119 (41.03)	132 (45.52)	251 (43.28)		
Very good	24 (8.28)	30 (10.34)	54 (9.31)		
**Attitude of health staff**					
I don't use it	9 (3.10)	7 (2.41)	16 (2.76)	8.608	0.126
Very bad	11 (3.79)	2 (0.69)	13 (2.24)		
Bad	22 (7.59)	15 (5.17)	37 (6.38)		
Neutral	87 (30.00)	90 (31.03)	177 (30.52)		
Good	131 (45.17)	141 (48.62)	272 (46.90)		
Very good	30 (10.34)	35 (12.07)	65 (11.21)		
**Convenience and accessible for service**					
I don't use it	8 (2.76)	16 (5.52)	24 (4.13)	9.518	0.090
Very bad	9 (3.10)	6 (2.07)	15 (2.59)		
Bad	20 (6.90)	10 (3.45)	30 (5.17)		
Neutral	94 (32.41)	79 (27.24)	173 (29.83)		
Good	134 (46.21)	146 (50.34)	280 (48.28)		
Very good	25 (8.62)	33 (11.38)	58 (10.00)		
**Cost and affordable for service**					
I don't use it	3 (1.03)	11 (3.79)	14 (2.41)	11.405	0.044
Very bad	15 (5.17)	5 (1.72)	20 (3.45)		
Bad	49 (16.90)	44 (15.17)	93 (16.03)		
Neutral	105 (36.21)	100 (34.48)	205 (35.34)		
Good	103 (35.52)	108 (37.24)	211 (36.38)		
Very good	15 (5.17)	22 (7.59)	37 (6.38)		
**Health technology and skill level**					
I don't use it	4 (1.38)	8 (2.76)	12 (2.07)	10.272	0.068
Very bad	5 (1.72)	4 (1.38)	9 (1.55)		
Bad	10 (3.45)	6 (2.07)	16 (2.76)		
Neutral	109 (37.59)	90 (31.03)	199 (34.31)		
Good	144 (49.66)	146 (50.34)	290 (50.00)		
Very good	18 (6.21)	36 (12.41)	54 (9.319)		

In terms of different dimensions, the biggest difference was observed in satisfaction with health service costs, which was also an aspect that new city citizens reported as improving the least after they obtained urban Hukou (Table 2, Figure 1).

### Healthcare Service Utilization and Expenditure

In terms of access convenience, there were no statistically significant differences between the groups for distance to the nearest health institution, with 65.0% of the participants living within one km of a basic healthcare institution. However, it appeared that the study group might spend more time traveling to these institutions (Table 3).

**Table 3:** Healthcare services utilization indicators of different group.

**Items**	**Type of citizen (%)**		**Total *N* = 580**	**χ^2^**	***p***
	**Control group *N* = 290**	**Study group *N* = 290**			
**Distance to nearest health institutions [km, kilo meter]** ^**a**^					
−1.0 km	118 (43.38)	104 (36.49)	222 (38.28)	8.917	0.112
−2.0 km	65 (23.90)	90 (31.58)	155 (26.72)		
−3.0 km	57 (20.96)	50 (17.54)	107 (18.45)		
−4.0 km	12 (4.41)	14 (4.91)	26 (4.48)		
−5.0 km	13 (4.78)	11 (3.86)	24 (4.14)		
5.0 km-	7 (2.57)	16 (5.61)	23 (3.97)		
**Traffic time to nearest health institutions [min, minute]** ^**b**^					
No visit	30 (10.34)	12 (4.41)	42 (7.24)	12.641	0.013
−15 min	194 (66.90)	199 (68.62)	393 (67.76)		
−20 min	40 (13.79)	36 (12.41)	76 (13.10)		
−30 min	16 (5.52)	30 (10.34)	46 (7.93)		
30 min-	10 (3.45)	13 (4.48)	23 (3.97)		
**Insurance coverage**					
EBMI	219 (75.52)	222 (72.76)	430 (74.14)	1.724	0.422
RBMI	63 (21.72)	74 (25.52)	137 (23.62)		
No BMI	8 (2.76)	5 (1.72)	13 (2.24)		
Commercial insurance	85 (29.31)	79 (27.24)	164 (28.27)	0.306	0.580
**Two-Weeks's clinic visit**					
Yes	106 (55.79)	94 (69.63)	200 (61.54)	6.387	0.011
No	84 (44.21)	41 (30.37)	125 (38.46)		
**Hospitalization utility**					
Yes	40 (13.79)	24 (8.28)	64 (11.03)	4.496	0.034
No	250 (86.21)	266 (91.72)	516 (88.97)		

*^a^The total was 557, as there were 18 aboriginal and 7 migrants failed to give the exactly distance.*

*^b^The total was 538, as there were 30 aboriginal and 12 migrants did not go the nearest health institutes*.

**Figure 2:**
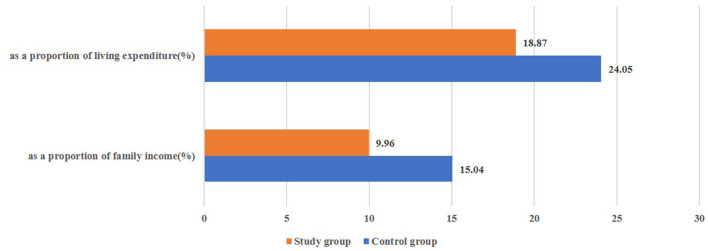
The proportion of annual health expenditures for the two groups (%).

In terms of affordability, 98.23% of new city citizens were covered by China's basic health insurance. Although the basic structure was similar, compared with cityward migrants, employment basic medical insurance (EBMI) coverage and commercial insurance ownership were higher among original residents (Table 3). Health expenditure was lower among the study group (5,000 RMB on average) than the control group (10,000 RMB on average, K-S Z = 1.62, *p* <0.05; Table 1, Figure 2).

The study group had a higher clinic visit utility rate and lower hospitalization utility rate than the control group (*p*_*clinic*−*visti*_ = 0.01*, p*_*hospitalization*_ = 0.03, Table 3).

The OLS regression analysis results showed that age, family size, marital status, living expenditure, and self-reported health status were all significantly related to health expenditure: on average, people who were older, married, had more family members, higher living expenditures, or lower health status, had higher health expenditure (Table 4). Furthermore, there were statistically significant differences regarding the factors that influence the two groups (Chow test F = 2.272, *p* = 0.003, Table 4, Appendix 6).

**Table 4:** Group regression model results for the impacts of actual health cost.

	**Total**	**Control group**	**Study group**
(Intercept)	−0.948 (−0.980)	−0.123 (−0.093)	−1.231 (−0.886)
Age	0.026** (4.804)	0.020* (2.390)	0.026** (3.433)
Family members	0.204** (4.927)	0.212** (3.961)	0.227** (3.593)
Family income#	0.078 (0.978)	−0.031 (−0.290)	0.214 (1.844)
Living expenditure#	0.540** (8.882)	0.612** (7.661)	0.426** (4.714)
[gender = 1]	0.004 (0.043)	−0.164 (−1.262)	0.145 (1.095)
[gender = 2]			
[marital status = 1]	0.429** (3.773)	0.323 (1.928)	0.483** (3.110)
[marital status = 2]			
[BHIS = 1]	0.547 (1.771)	0.896* (2.325)	0.059 (0.123)
[BHIS = 2]	0.601 (1.902)	1.097** (2.745)	−0.028 (−0.059)
[BHIS = 3]			
[employee = 1]	−0.131 (−0.621)	−0.419 (−1.347)	0.106 (0.376)
[employee = 2]	−0.215 (−0.796)	−0.137 (−0.339)	−0.318 (−0.856)
[employee = 3]			
[education = 1]	−0.042 (−0.256)	0.207 (0.841)	−0.089 (−0.407)
[education = 2]	0.002 (0.019)	0.301 (1.966)	−0.266 (−1.597)
[education = 3]			
[Health status = 1] 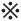	0.437** (3.237)	0.515** (2.748)	0.285 (1.458)
[Health status = 2] 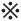	0.443** (3.798)	0.467** (2.733)	0.370* (2.349)
[Health status = 3] 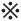			
[commercial insurance = 1]	0.148 (1.416)	0.244 (1.711)	0.065 (0.433)
[commercial insurance = 2]			
N	464	216	248
*R* ^2^	0.31	0.43	0.281
Adjust *R*^2^	0.286	0.388	0.234
F	*F*_(15, 448)_ = 13.394, *p* = 0.000	*F*_(15, 200)_ = 10.079, *p* = 0.000	*F*_(15, 232)_ = 6.044, *p* = 0.000
White's test	χ^2^ = 133.110, *p* = 0.147	χ^2^ = 125.767, *p* = 0.116	χ^2^ = 121.698, *p* = 0.229

Dependence [actual health cost], Link function: OLS; * p <0.05 ** p < 0.01.

*Transformed data were actual health cost, family income, family expenditure, and self-report health status.*

*# Data transformed method: ln; 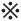 Data transformed method: self-report health status was cast into three level by organizing the value into quartile ranges (self-report health status values in [~10^th^ percentiles] =health status 1, self-report health status values in [90^th^ percentiles ~] =health status 3, else =health status 2)*.

## Discussion

This study provided a basic categorical review of the use of health services among the large cityward migrant population to help authorities and researchers understand the process and improve basic human rights during China's urbanization.

### Improvements in Migrant Residents' Health

Generally, the results of this study indicated an improvement in health status for migrant citizens. The health status of new city citizens was better than the control group, and the self-reported illness rate was lower for them than for original residents, which proved that the rural-to-urban movement provided a significant driving-force for promoting the health of migrant groups (19). The prevalence of chronic diseases was 13% lower than that of the urban level in central China (Appendix 7) (18). Meanwhile, working in cities was a strong influence on the sample selected of rural-to-urban immigrants (20) and the migrant new city citizen residents were in good health (6). While health status was lower than average in urban areas during the same period (78.7 in central China) (18), the self-reported health status of new city citizens was better (74.6 on average, 0, 100) compared with those in rural areas (74.4 in central China, 2018, Appendix 7) and among original residents with the same demographic conditions (Table 1, Appendix 7). Previous studies demonstrated that working in cities required a higher health status; therefore, migrant laborers were generally younger and healthier than those who remained in the countryside (2, 10, 21), which was confirmed in our study. Likewise, the results of the present study indicated that the migrant group included mainly middle-aged people with the ability to work, and with better health status than those in the control group, as well as rural people.

Specifically, access to basic health services for the new city citizens improved after obtaining urban Hukou (Figure 2), and there were few differences in the fairness of the provision of health services, except for cost and affordability (Table 3). This may be due to China's ongoing comprehensive policy of ensuring equitable and homogenous health services for different groups, particularly in major cities, such as Beijing (22), Shenzhen (23), Guangzhou (24), and Wuhan (25).

Moreover, the frequency of 2-weeks' clinical visits was significantly higher for the study group than the control group and the national average (Appendix 7), indicating that with the accumulation of wealth and convenience of information dissemination during urbanization, health service needs increased (6, 10, 14, 26). This was closely correlated with the continuous improvement of health insurance policies. The basic health insurance coverage rate was 98.3% for the study group. As most of the surveyed people were workers, EMBI coverage was higher than the national average. Moreover, this reflected the growing consideration of migrant groups toward health maintenance. Over 70% of the respondents demonstrated self-medication behavior, although 53.45% of the migrants reported no illnesses, which was 19% higher than the control group. The OLS results showed no significant disparity in the different kinds of insurance coverage for migrated citizens.

### Aspects Requiring Further Attention

Although there have been significant improvements in the availability and use of health services, the possibilities of health inequities still exist. The results of this study showed that migrant residents lived within 2 kms (68.07%) and 15 min (67.76%) of the nearest health institution, which was slightly more inconvenient than the national average during the same period (85.5 and 91.6%, respectively, Appendix 7). This may be due to unfamiliarity with the living environment, resulting in a lack of knowledge of the surrounding community health facilities. However, it is more likely that the local public service facilities are insufficient in the areas where new citizens congregate (27).

Furthermore, the cost of health is an important factor for the health maintenance of new city citizens. Although the migrant residents had higher income and consumption levels than the local annual average (Appendix 1), the health expenditure of this group was lower than the control group; affordability showed the least improvement and was the only dimension with a difference in satisfaction. The OLS results showed that the influencing factors for health expenditure included age, family size, marital status, family living expenditure, and self-reported health status. Further, differences in coefficients (*F* = 2.272, *p* = 0.003, Table 4, Appendix 6) imply that migration and urbanization are accompanied by significant structural disparities in factors influencing health expenditure.

With increased health consumption and expenditure, there is greater pressure on health service delivery and the operation of basic health insurance funds. Commercial insurance could alleviate the disease burdens of migrant residents (28) until the accumulation of wealth and social networks are equivalent to that of original residents (11). However, our results show that the coverage and efficiency of commercial insurance is still far from ideal, with <30% participants possessing commercial insurance.

### Different Factors Influencing Health Service Utilization

The disparities can be explained as follows: Aging has become a pertinent societal issue, particularly in relation to urbanization (29, 30). The OLS results of this study indicate that the healthcare needs of elderly migrants moving with their families, was found to be significantly related to family health expenditure, only for migrant new citizens (Table 4). This indicates that for elderly migrants, the lack of relationship transfers and the renewing of social security systems, besides health insurance, might lead to greater pressure on families in terms of healthcare expenditure.

Furthermore, differences in previous identities may influence cognitive awareness and other perspectives on health, which could lead to different choices in health utilization. After PSM, the demographic situation of the two groups was essentially the same; however, there were significant differences in educational background. There were more migrants with a middle school education and below than those in the control group. This mirrors the educational level of the floating groups reported in previous studies (6, 24), which likely affects health consciousness (9) and health service utility (9, 23). The educational difference may affect cognitive awareness and other aspects of health, potentially leading to different choices in health utilization. Traditional culture influences health consciousness, and has a significant effect on the health choices of migrant residents (31). Family plays a vital role in traditional Chinese social culture (32). After obtaining the urban Hukou, the new city citizens group settled in urban areas with their families (the average family size was three members) (18) and the cost of living increased compared with that of floating citizens (the average family scale was reported to be 2.3 in Beijing, 2.6 in Guangzhou, and 1.6 in Chongqing) (22, 24). Even with the increases in health insurance coverage and family income alleviating disease burden and improving the risk resistance of new city citizens (19), married people with more family members required greater coverage, thus increasing the pressure and costs of health maintenance. Fortunately, our survey reflected that the critical gender gap in health consumption had been vanishing in the urbanization process, with previous study pointed that woman being the vulnerable group in terms of health services utility (33). However, the interaction of different demographic factors and health behaviors could be investigated in depth.

In addition, our survey showed that health expenditure differed between inpatient and outpatient medical services. National statistics indicated that hospitalization costs in China (34) were significantly higher than outpatient costs (33 times higher for hospitals, and 24 times higher for community health institutions in 2018, Appendix 8). The findings in this study showed that the utilization of inpatient services by new city citizens was significantly lower than outpatient services, and the utilization of inpatient services by the control group was higher than for new city citizens. This could provide a good explanation for the differences in actual health expenditure between the two groups.

This study has some limitations. First, recall bias may have influenced self-reported illnesses and treatment-seeking behavior (24). Second, although this study addressed medical services, it did not discuss public health services, mental health, and other related health dimensions, which might also influence health behaviors and consumption. Third, the sample area targeted by the study was only the central region of China, which is not representative of the entire country. Finally, the investigation of the influencing factors for health service utilities by new city citizens was shallow and lacked in-depth analysis. Furthermore, the effect and persuasiveness of cross-sectional studies may be limited. Therefore, in the future, cohort studies should conduct ongoing follow-up surveys of new citizen groups.

## Conclusions

Urbanization continues to attract people to cities, and results in changing lifestyles, economic resources, and health. Migrant groups enjoy improved primary public services, such as health services. The new city citizens are a group driven by “healthy choices”, and are generally in good health. Obtaining urban Hukou helps meet their health service needs and brings equal access to health services. However, even after this identity shift, migrant residents still face great pressure regarding health consumption, which should be addressed by policy makers and local administrators.

The influx of rural residents into cities drives urbanization in China. The increasing number of immigrants increases the labor pool of cities, while putting pressure on many areas, including healthcare. This study highlighted the need for active primary healthcare and integration of the health service system, particularly services for the elderly, women, and families (24, 35). In addition, healthcare strategies should be adjusted, while complementary commercial insurance should be promoted (28). Furthermore, healthcare policies and knowledge should be explained to immigrant families in detail (6, 20) to improve their health awareness and alleviate the pressure of health consumption.

## Data Availability Statement

The data analyzed in this study is subject to the following licenses/restrictions: data collected from yearbooks are available in the reports listed in the references. Data were collected from residents according to the statistical law of the People's Republic of China. Personal information was erased after the confidentiality agreement between the research team and interviewees was obtained. The research team maintained the security of the initial data. Data are available from the corresponding author upon reasonable request. Requests to access these datasets should be directed to PF, pfang@mails.tjmu.edu.cn; RM, ruimin0801@163.com.

## Author Contributions

PF planned the study. PF, RM, and CZ substantially contributed to the acquisition, analysis, and interpretation of data. ZF and CT mainly contributed to the conception and design. RM and PF contributed to coordination of data collection. All authors contributed substantially to the interpretation of the results and writing of the manuscript and read and approved the final version of the manuscript.

## Funding

This study was funded by grants from the Fundamental Research Funds for the Central Universities (2020kfyXJJS057) and the China Medical Board-Opening Competition program 2015 (No. CMB15-223). The sponsors played no role in the design and implementation of the study, collection, management, analysis, and interpretation of the data, preparation, review, and approval of the manuscript, and decision to submit the manuscript for publication. This research received considerable assistance from A/Prof. Xingjie Hao, and Lec. Peng Wang with the statistics and data analysis, and help from Mrs. Li Zhang, Mrs. Zhihong Ding, Mr. Min Zhang for the field survey.

## Conflict of Interest

The authors declare that the research was conducted in the absence of any commercial or financial relationships that could be construed as a potential conflict of interest.

## Publisher's Note

All claims expressed in this article are solely those of the authors and do not necessarily represent those of their affiliated organizations, or those of the publisher, the editors and the reviewers. Any product that may be evaluated in this article, or claim that may be made by its manufacturer, is not guaranteed or endorsed by the publisher.

## References

[1] Asian Development Bank. Addressing climate change and migration in Asia and the Pacific. Metro Manila, Philippines (2012).

[2] Khan MMH Kraemer A. Are rural-urban migrants living in urban slums more vulnerable in terms of housing, health knowledge, smoking, mental health and general health? Int J Soc Welf. (2014) 23:373–83. 10.1111/ijsw.12053

[3] Fay M. The urban poor in Latin America. Washington, DC: The World Bank (2005). 10.1596/0-8213-6069-8

[4] Kwankye SO Richter S Okeke-Ihejirika P Gomma H Obegu P Salami B. A review of the literature on sexual and reproductive health of African migrant and refugee children. Reprod Health. (2021) 18:81. 10.1186/s12978-021-01138-3 33865417 PMC8052768

[5] Onwujekwe O Mbachu CO Ajaero C Uzochukwu B Agwu P Onuh J. Analysis of equity and social inclusiveness of national urban development policies and strategies through the lenses of health and nutrition. Int J Equity Health. (2021) 20:101. 10.1186/s12939-021-01439-w 33863330 PMC8051828

[6] Gong P Liang S Carlton EJ Jiang Q Wu J Wang L. Urbanisation and health in China. Lancet. (2012) 379:843–52. 10.1016/S0140-6736(11)61878-3 22386037 PMC3733467

[7] National Bureau of Statistics of China. China Statistical Yearbook. Beijing: China Statistics Press (2020).

[8] Ortigoza AF Tapia Granados JA Miranda JJ Alazraqui M Higuera D Villamonte G. Characterising variability and predictors of infant mortality in urban settings: findings from 286 Latin American cities. J Epidemiol Commun Health. (2021) 75:264–70. 10.1136/jech-2020-215137 33060193 PMC7892385

[9] Salazar MA Hu X. Health and lifestyle changes among migrant workers in China: implications for the healthy migrant effect. Lancet Diabetes Endocrinol. (2016) 4:89–90. 10.1016/S2213-8587(15)00438-6 26776862

[10] Hu X Cook S Salazar MA. Internal migration and health in China. Lancet. (2008) 372:1717–9. 10.1016/S0140-6736(08)61360-4 18930533 PMC7135200

[11] Shao S Wang M Jin G Zhao Y Lu X Du J. Analysis of health service utilization of migrants in Beijing using Anderson health service utilization model. BMC Health Serv Res. (2018) 18:462. 10.1186/s12913-018-3271-y 29914464 PMC6006712

[12] Central People's Government of the People's Republic of China. National New Urbanization Plan of China (2014–2020). (2014). Available online at: http://www.gov.cn/zhengce/2014-03/16/content_2640075.htm.

[13] Lei K Li L. Discrimination against rural-to-urban migrants: the role of the Hukou system in China. PLoS ONE. (2012) 7:e46932. 10.1371/journal.pone.0046932 23144794 PMC3489849

[14] Mou J Griffiths SM Fong HF Dawes MG. Defining migration and its health impact in China. Public Health. (2015) 129:1326–34. 10.1016/j.puhe.2014.01.010 25515044

[15] National Development and Reform Commission of China. Notice on the issuance of the National Comprehensive Pilot Program for New Urbanization (2015). Available online at: http://www.gov.cn/xinwen/2015-02/04/content_2814341.htm.

[16] Enders CK. Using the expectation maximization algorithm to estimate coefficient alpha for scales with item-level missing data. Psychol Methods. (2003) 8:322–37. 10.1037/1082-989X.8.3.322 14596494

[17] Cheema JR. Some general guidelines for choosing missing data handling methods in educational research. JMASM. (2014) 13:53–75. 10.22237/jmasm/1414814520

[18] National Center for Health Statistics and Information. An analysis report of national health servcie survey in China. (2018). Beijing: People's Health Press (2021).

[19] Yang G Wang Y Zeng Y Gao GF Liang X Zhou M. Rapid health transition in China, 1990–2010: findings from the Global Burden of Disease Study 2010. Lancet. (2013) 381:1987–2015. 10.1016/S0140-6736(13)61097-1 23746901 PMC7159289

[20] Yan Li. Understanding health constraints among rural-to-urban migrants in China. Qual Health Res. (2013) 23:1459–69. 10.1177/1049732313507500 24122513

[21] Chen J. Internal migration and health: re-examining the healthy migrant phenomenon in China. Soc Sci Med. (2011) 72:1294–301. 10.1016/j.socscimed.2011.02.016 21435765

[22] Floating Population Services and Management Division. Report on China's migrant population development 2016. Beijing: National Population and Family Planning Commission of P.R.C (2016).

[23] Lam KKF Johnston JM. Health insurance and healthcare utilisation for Shenzhen residents: a tale of registrants and migrants? BMC Public Health. (2012) 12:868. 10.1186/1471-2458-12-868 23061720 PMC3584814

[24] Song X Zou G Chen W Han S Zou X Ling L. Health service utilisation of rural-to-urban migrants in Guangzhou, China: does employment status matter? Trop Med Int Health. (2016) 22:82–91. 10.1111/tmi.12801 27775826

[25] Health Family Planning Commission of Wuhan. Notice on Issuing the Implementation Plan of Wuhan Floating Population Health Education Promotion Action. (2017). Available online at: http://www.wuhan.gov.cn/zwgk_28/zc/qtbmwj/202001/t20200114_803354.shtml.

[26] Li X Song J Lin T Dixon J Zhang G Ye H. Urbanization and health in China, thinking at the national, local and individual levels. Environ Health-Glob. (2016) 151:S32. 10.1186/s12940-016-0104-5 26961780 PMC4895783

[27] Chen Y Wang B Liu X Li X. Mapping the spatial disparities in urban health care services using taxi trajectories data. Transactions in GIS. (2018) 22:602–15. 10.1111/tgis.12333

[28] Liu J Chen H Chen Y Li Z. Exploring the relationship between migrants' purchasing of commercial medical insurance and urbanisation in China. BMC Health Serv Res. (2018) 18:679. 10.1186/s12913-018-3503-1 30176868 PMC6122700

[29] Phillipson C. Urbanisation and ageing: towards a new environmental gerontology. Ageing Soc. (2004) 24: 963–72. 10.1017/S0144686X04002405

[30] Beard JR. Ageing and urbanization: can cities be designed to foster active ageing? Public Health Reviews. (2010) 32:427–50. 10.1007/BF03391610

[31] Ye X Zhu D He P. Earlier migration, better cognition? the role of urbanization in bridging the urban-rural cognition gaps in middle and older age. Aging and Mental Health. (2021)3:1–9. 10.1093/ije/dyab168.271 33467900

[32] Fei N. Study on family migration in the process of urbanization: patterns and dilemma: based on cases of two generation migrant workers' families (only Chinese version). Theory Monthly. (2021) 4:134–43. 10.14180/j.cnki.1004-0544.2021.04.015

[33] Vlassoff C. Gender differences in determinants and consequences of health and illness. J Health Popul Nutr. (2007) 25:47–61. 17615903 PMC3013263

[34] China National Health Commission (NHC). China Health Statistical Yearbook. Beijing: China Union Medical University Press (2020).

[35] Liu D Meng H Dobbs D Conner KO Hyer K Li N. Cross-sectional study of factors associated with community health centre use in a recently urbanised community in Chengdu, China. BMJ OPEN. (2017) 7:e014510. 10.1136/bmjopen-2016-014510 28600364 PMC5541612

