# Supplementary material for: Do Migrant Residents Really Achieve Health Equity by Obtaining Urban Hukou? A Comparative Study on Health Service Utilization and Urbanization in Central China
Source: Front Public Health. 2022 Apr 5;10:784066. doi: 10.3389/fpubh.2022.784066 (PMC9037327; doi:10.3389/fpubh.2022.784066)
Supplement: Supplementary file 1 [file Data_Sheet_1.pdf]

Appendix 1 Brief introduction of the research area

Hubei Province is located in the south-central of mainland China, the midstream of the Yangtze River(location map), the central part of *Jianghan* Plain, one of the three important planting areas, with a long history of civilization. With over 58.85 million resident population, Hubei kept the top 1 urbanization rate in 6 provinces of Central China. At the end of 2017, the urban population of Hubei Province was 35 million, and the urbanization level in Hubei Province was 59.3% in 2017, which is higher than the national average. Also, Hubei had provincial capital-level, city-level and county-level pilot pots of China's registration reform, which launched in 2014 as the first batch[1].

Wuhan City is the capital city of Hubei Province and national center city, with the first place in population, economy and social development in Hubei. In 2014, Wuhan was approved as one of the first batch of national pilot projects for new-type urbanization

Located in the northwest of Hubei Province, Xiangyang City was the other one of Hubei's two pilots for the 1st batch of national pilot projects for new-type urbanization. In June 2015, Xiangyang City launched the most thorough household registration reform in China.

The basic information was as following tables:

Appendix-Table 1 Urbanization rate of different level

	National level	Provincial level	Wuhan city	Xiangyang city
**2010**	50.0%	49.7%	77.1%	47.3%
**2014**	54.8%	55.7%	79.4%	56.0%
**2017**	58.5%	59.3%	80.0%	59.7%

Appendix-Table2 Living population in urban areas (*10^6)

	National level	Provincial level	Wuhan city	Xiangyang city
**2010**	665.6	28.5	5.4	2.6
**2014**	749.2	32.4	5.6	3.1
**2017**	813.5	35.0	8.7	3.4

Appendix-Table3 Per capita disposable income (Chinese Yuan, RMB)

	National level	Provincial level	Wuhan city	Xiangyang city
**2010**	19,109	16,058	20,806	13,333
**2014**	28,844	24,852	33,270	24,113
**2017**	36,396	31,889	43,405	31,316

[1] National Development and Reform Commission of China. Notice on the issuance of the National Comprehensive Pilot Program for New Urbanization[Z]. 2015-2-14: 2021-5-20.

Appendix-figure 1 Location of Hubei province

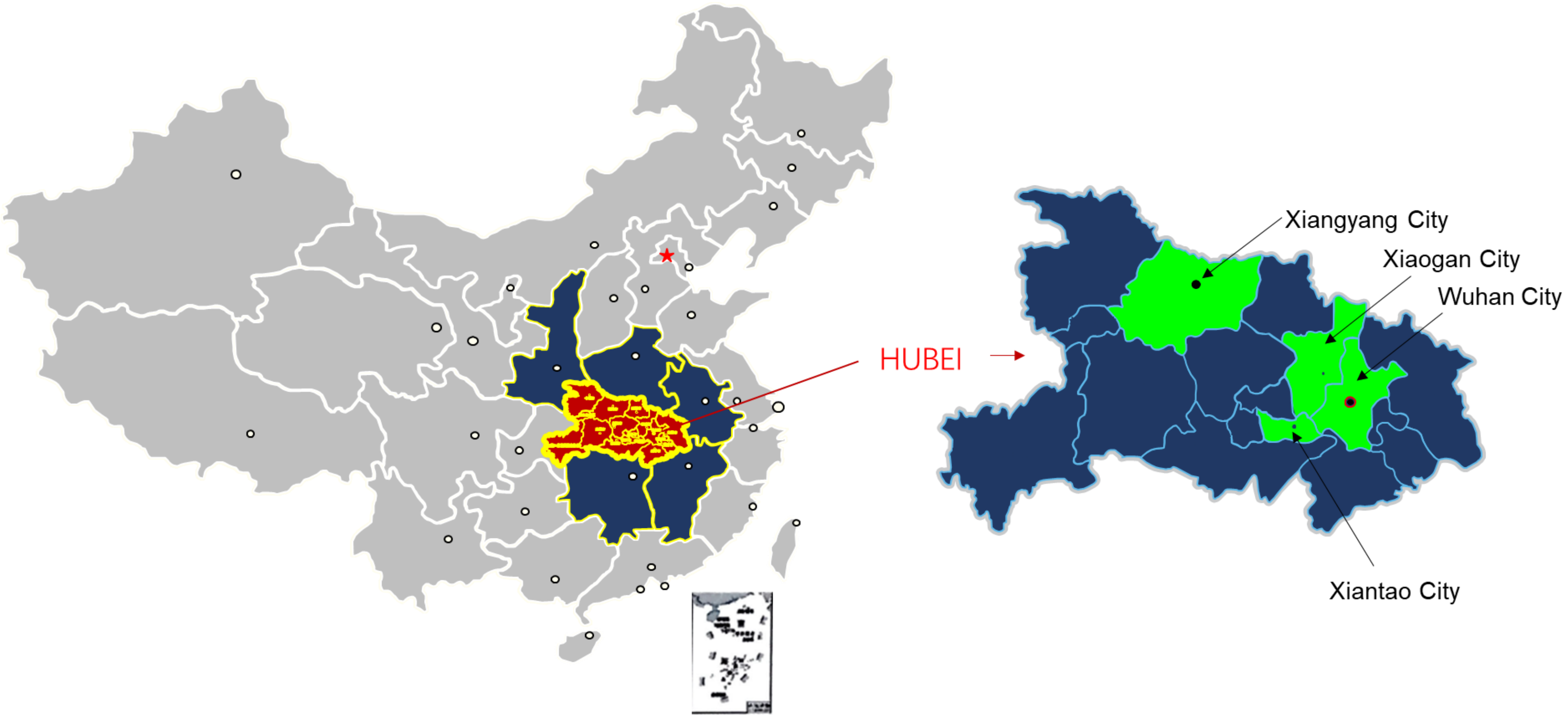

Note： a) The left map showed the geographic location of Hubei province in China, and the right map showed the administrative divisions of Hubei province.

b) The green shadow showed the pilot pots of the first batch of national comprehensive pilot projects for new urbanization in Hubei province, launched in 2014 by the General Office of National Development and Reform Commission

Appendix 2 The sampling process

According to the Chinese General Social Survey 2015 data, the mean of satisfaction feeling in health service for urban residents was 67.41±17.98, and for migrated residents (group1) was 71.30, while for aboriginal residents (group2) was 67.15. The sampling ratio was 2:1. Usually, the type I error rate was 5% at 2-sided test, and the type II error rate was 10%-20% in group-designed studies [2,3]

$$n=\frac{(q_1^{-1}+q_2^{-1})(t_{\alpha/2}+t_{\beta})^2 S^2}{\delta^2}$$

$$\alpha = 0.05, \beta = 0.1, 1-\beta = 0.9,$$

$$\text{sample ratio}=q_1 : q_2 = 2:1$$

The estimated sample was 869 people. After the export group discussion and easy to carry out, the research sample was set as 900 (600 for new settled residents, and 300 for aboriginals). And the whole sampling process for study analysis was as followed flow chart:

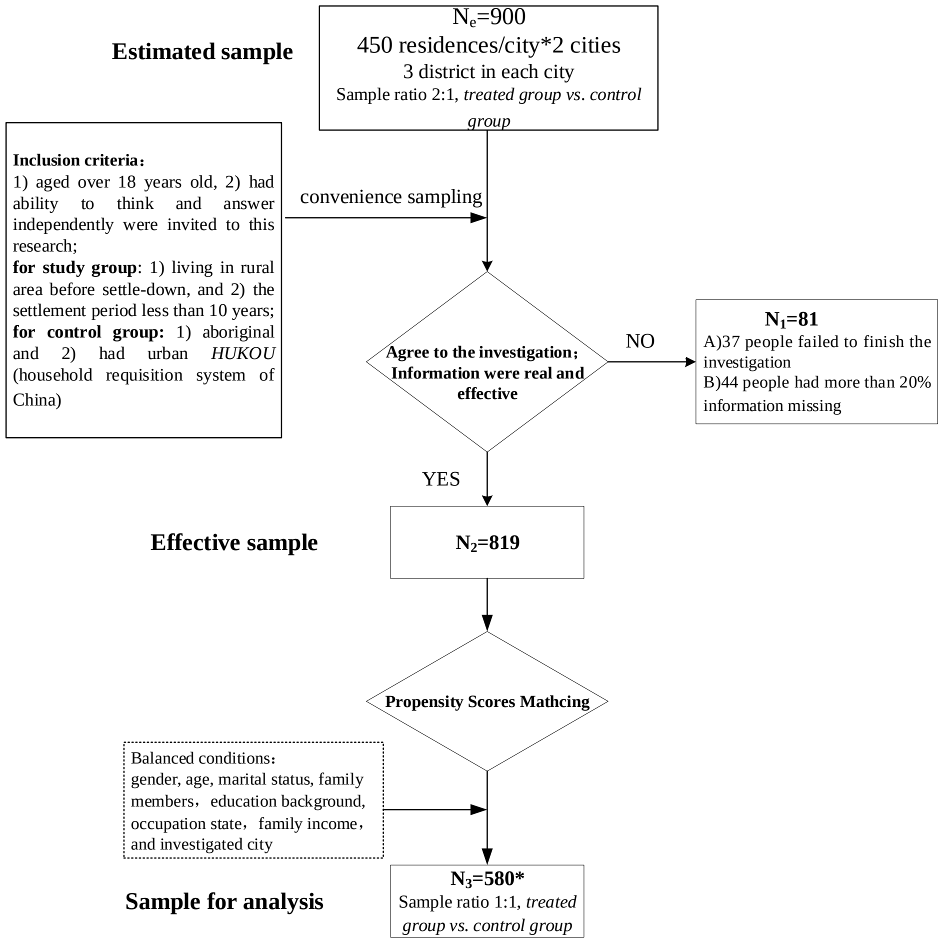

Appendix-figure 2 flow chart for analysis sample selection.

Note: 2 Chow S, Shao J, Wang H. 2008. *Sample Size Calculations in Clinical Research*. 2nd Ed. Chapman & Hall/CRC Biostatistics Series. page 58.

3 The formulas and calculators were completed in the website of *'power and sample size*'. Available at http://powerandsamplesize.com/Calculators/Compare-2-Means/2-Sample-Equality

* According to the sample size calculation formula, at the 1:1 sample level, when the actual sample size was 580, the power was 0.9 after check.

Appendix 3 Questionnaire (Chines version and translations)

问卷编号：□□□□□□

【调查员不需填写问卷编号】

市民医疗服务情况调查问卷（湖北省）

调查员签名:	复核员签名:
受访人类型：新市民 □ 普通市民 □<br>（新市民为 2007 年以来在调查地点新落户为城镇居民的农业转移人口，排除学校集体户口者和无自主作答能力者；普通市民为自 2000 年起即为本市城镇户口的市民。）	
调查地点：_______省_______市________区	
调查时间：2018 年□□月□□日	

尊敬的女士/先生：

您好！感谢您阅读这份调查问卷。此问卷是为了了解我国新型城镇化进程中市民的健康状况和医疗服务利用情况而设计的。调查员将询问您的个人情况、医疗卫生服务情况以及一些经济生活情况。如果给您的生活和工作造成不便，我们深表歉意，请您理解和支持我们的工作。

本次调查收集的所有资料仅供研究之用，我们将按照《统计法》的规定，严格保密，不会以任何形式公开您的个人信息。所有问题的回答均无对错之分，请您根据实际情况回答访问员提出的问题。

感谢您对我们工作的支持！

第一部分 基本信息	
101	您的出生日期【　　】年【　　】月（填写 4 位年份，2 位月份）
102	您的性别【　　】 ①男　　②女
103	您的婚姻状况【　　】<br>①在婚　　②单身（包括未婚、丧偶、离婚）
104	您的文化程度【　　】<br>①初中及以下　②高中/技校/中专　③本科、大专或以上
105	您的就业状况【　　】<br>①在业(包括灵活就业）　②离退休<br>③在校学生**（停止作答）**　④失业、无业**（跳至 108）**
106	您在本市已经居住了【　　】年【　　】月（指时间长度）
107	您目前的户口状况【　　】<br>①本市城镇居民户口　②本市农村居民户口<br>③本市以外的农村居民户口　④本市以外的城镇居民户口<br>**（若回答②③④，则停止答题）**
108	您在落户本市城镇户口之前的户口状况【　　】<br>①本市农村居民户口　②本市以外的农村居民户口　③本市以外的城镇居民户口
109	去年，您的家庭年总收入是【　　】
110	您的家庭人口数（指共同负担开支的人口）【　　】
111	去年，您家全部的生活消费性支出为多少元？【　　】(除各类投资以外)
112	去年，您家在药品、医疗、保健支出总计多少元？【　　】
113	离您家最近的医疗机构有多少公里？【　　】(医疗机构包括社区卫生服务中心/站，卫生院、村卫生室、诊所、综合医院和中医医院等)<br>①不足 1 公里　② 1～2 公里　③ 2～3 公里<br>④ 3～4 公里　⑤ 4～5 公里　⑥ 5 公里及以上
114	从您家到最近医疗机构需要【　　】分钟？（以步行或最容易获得或搭乘的交通工

	具的最快方式)
**第二部分 个人健康状况**	
201	与同龄人相比，您认为您的健康状况如何？【 】<br>├---┼—-┼—-┼—-┼—-┼—-┼—-┼—-┼—-┼—-┤<br>0 10 20 30 40 50 60 70 80 90 100<br>最差健康状况 最好健康状况
202	调查前 6 个月之内，您是否患有经医生确诊的慢性病？【 】(包括以下两种情况：第一，调查前半年内，经过医务人员明确诊断的慢性病；第二，调查半年以前患有医生诊断的慢性病，在调查前半年内时有发作并采取了治疗措施如服药、理疗，或者一直在治疗以控制慢性病的发作等。)<br>①是 ②否 ③不清楚
203	调查前 14 天（2 周）内您的身体是否有过伤病或不适（包括生病或受伤）【 】<br>①是 ②否（跳至 301）
204	您感到不适后，是否自行购药/服药【 】①是 ②否
**第三部分卫生服务利用**	
301	您目前所参加的基本医疗保险形式是【 】<br>①城镇职工基本医疗保险 ②城乡居民基本医疗保险<br>③公费医疗 ④都未参加
302	目前，您是否拥有商业健康保险？【 】 ①是 ②否
303	您两周内是否因伤病到医疗机构就诊？【 】 ①是**（跳至 305）** ②否
304	您两周内未就医的原因【 】**（结束后跳至 307）**<br>①两周前就医，遵医嘱持续治疗中 ②自感病轻 ③经济困难 ④就诊麻烦<br>⑤无时间 ⑥交通不便 ⑦无有效措施 ⑧其他<br>[ ]
305	去年，您是否住过院【 】<br>①住过 ②未住过**（若选②，则跳到第 401 题）**
**第四部分 医疗服务体验感受及满意度评价**	

	很不满意	不太满意	一般	比较满意	非常满意	没去过不了解

401	总体而言，您对目前生活状态的评价是	1	2	3	4	5	0
402	您对目前居住地医疗技术水平的评价	1	2	3	4	5	0
403	您对目前居住地医疗服务费用的评价	1	2	3	4	5	0
404	您对目前居住地看病方便程度的评价	1	2	3	4	5	0
405	您对目前居住地医护人员医疗服务态度的评价	1	2	3	4	5	0
406	您对目前居住地医疗服务体系的总体评价	1	2	3	4	5	0
**与落户城镇之前相比**<br>**（407-412 由新市民回答）**		**大幅改善**	**略有改善**	**没有变化**	**略有恶化**	**大幅恶化**	**没去过不了解**
407	您的生活状态的总体变化	1	2	3	4	5	0
408	您觉得医疗技术水平有什么变化	1	2	3	4	5	0
409	基本医疗保险的报销程度，使您的经济状况	1	2	3	4	5	0
410	您在看病方便程度方面有什么变化	1	2	3	4	5	0
411	医护人员医疗服务态度的变化	1	2	3	4	5	0
412	您目前获得的医疗服务情况总体来说	1	2	3	4	5	0

Translations

Questionnaire ID：□□□□□□

Medical Service Questionnaire for Hubei Province

Investigators：	Reviewer：
The type of interviewee： New city citizens □ ordinary citizens □<br>(The new city citizens are the agricultural transfer population who have settled in urban and got urban HUKOU since2007, excluding the school collective hukou and those who are unable to answer independently; the ordinary citizens are residents had the urban HUKOU since born.)	
Location of survey： _______Provincial_______City_______ District_________________________	
Survey time： 2018.□□.□□	

Dear Lady/Sir:

Hello! Thank you for reading this questionnaire. This questionnaire is designed to understand the health status of citizens and the utilization of medical services in the process of new urbanization in China. Investigators will ask about your personal situation, feelings for medical services, and some financial information. We apologize for any inconvenience to your life and work, please understand and support our work.

All information collected in this survey is for research purposes only and we will keep your personal information confidential in strict accordance with the provisions of the Statistics Law and will not disclose your personal information in any form. There is no right or wrong answer to all questions, please answer the questions raised by the visitor as they are.

Thank you for your support of our work!

**Part 1 Basic information**	
101	Date of birth【　　】year【　　】month
102	gender【 】 ① male ② female
103	Your marital status【　　】<br>①married ②Single (including unmarried, widowed and divorced)
104	Your educational level【　　】<br>① Junior high school and below ② senior high school / technical school / technical secondary school ③ undergraduate, college or above
105	Your employment status【　　】<br>① Employment (including flexible employment) ② retirement<br>③ School students (stop answering) ④ unemployed and unemployed (jump to 108)
106	How long have you lived in this city? 【　　】years (refer to the length of time)
107	Your current HUKOU status【　　】<br>① Registered permanent residence of urban residents in this city ② registered permanent residence of rural residents in this city<br>③ Registered permanent residence of rural residents outside the city ④ registered permanent residence of urban residents outside the city<br>(if answer ② ③ ④, stop answering the question)
108	Your HUKOU status before you settled in the city's urban account【　　】<br>① Registered permanent residence of rural residents in the city<br>② Registered permanent residence of rural residents outside the city<br>③ Registered permanent residence of urban residents outside the city
109	Last year, your family total annual income was【　　】
110	Your family population (refers to the population sharing expenses)【　】
111	Last year, how much was your family total living consumption expenditure?<br>【　　】(except for various investments)
112	Last year, how much did your family spend on medicine, medical treatment and health care? 【　　】
113	How many kilometers is the nearest health institution to your home?【　　】(Health institutions includes community health service centers / stations, health centers, village clinics, clinics, general hospitals and traditional Chinese medicine hospitals, etc.)<br>① Less than 1km ② 1 ~ 2km ③ 2 ~ 3km ④ 3 ~ 4km ⑤ 4 ~ 5km ⑥ 5km and above
114	It takes 【　　】minutes from your home to the nearest medical institution（The fastest way to walk or the most easily accessible or accessible means of transport)
**Part 2 Personal health status**	
201	What do you think of your health status compared with your peers?【　　】<br>├ ┼ ┼ ┼ -┼ ┼ ┼ ┼ ┼ ┼ ┤

	0 10 20 30 40 50 60 70 80 90 100<br>Worst Best
202	Have you suffered from chronic diseases confirmed by your doctor within 6 months before the survey? 【 】 (It includes the following two situations: first, chronic diseases clearly diagnosed by medical staff within the first half of the survey; second, the chronic diseases diagnosed by doctors six months ago were investigated, and they occurred from time to time within the first six months of the investigation, and treatment measures were taken, such as medication, physiotherapy, or continuous treatment to control the onset of chronic diseases.)<br>① Yes ② No ③ Unclear
203	Have you had any injuries or discomfort (including illness or injury) in the 14 days (2 weeks) before the investigation 【 】<br>① Yes ② No **（skip to 301）**
205	If you feel unwell, do you purchase / take medicine by yourself 【 】 ① Yes ② No
**Part 3 utilization of health services**	
301	What kind of basic health insurance do you have? 【 】<br>① Basic insurance for urban employees ② basic insurance for urban and rural residents<br>③ Public medical treatment ④ I did not participate
302	Currently, do you have commercial health insurance? 【 】 ① Yes ② no
303	Do you go to a medical institution for treatment due to injury within two weeks? 【 】 ① Yes **（skip to 305）** ② No
304	Reasons for your failure to see a doctor within two weeks 【 】 **（skip to 306）**<br>① See a doctor two weeks ago and continue treatment according to the doctor's advice<br>② mild self-infection ③ financial difficulties ④ trouble in seeing a doctor<br>⑤ No time ⑥ inconvenient traffic ⑦ no effective measures ⑧ others
305	Did you live in the hospital last year 【 】 ①yes ②no **（skip to 401）**

Part 4 The medical service experience and satisfaction evaluation

		**Very bad**	**Bad**	**Neutral**	**Good**	**Very good**	**I don't use it**
401	your feelings of the medical technology level currently	1	2	3	4	5	0
402	your feelings of the medical cost currently	1	2	3	4	5	0
403	your feelings of the convenience for health service currently	1	2	3	4	5	0
4	your feelings of the attitude of medical	1	2	3	4	5	0

04	staff currently						
4 05	your general feelings on health service currently	1	2	3	4	5	0
**Compared with before settling in town（406-410 only for new city citizens）**		**Much worse**	**Worse**	**No change**	**better**	**Much better**	**I don't use it**
4 06	Changes in medical technology level currently	1	2	3	4	5	0
4 07	The reimbursement degree of basic medical insurance makes your economic situation	1	2	3	4	5	0
4 08	Changes in the convenience of seeing a doctor	1	2	3	4	5	0
4 09	Changes in attitude of medical staff	1	2	3	4	5	0
4 10	Generally, changes in health service	1	2	3	4	5	0

Appendix 4 Study indicators

Indicators	Type
**1) Basic demographic information**	
**Gender**	[categorical variable]<br>1=male, 2=female
**Age**	[continuous variable]
**Marital status**	[categorical variable]<br>1= married, 2= single/ widowed/ divorced
**Education background**	[categorical variable]<br>1=middle school and below, 2=high school or technical college, 3=bachelor and above
**Occupation status**	[categorical variable]<br>1=employed, 2=retired, 3=unemployed or else
**Family members**	[categorical variable]<br>1= '1 people', 2= '2-4 people', 3= '>4 people'
**Registration information**	[categorical variable]<br>1=control group (Aboriginal), 2= Treated group (Migrant)
**Annul family income**	[continuous variable]
**Living expenditure**	[continuous variable]
**2) Health statues**	
**Self-report health status**	[continuous variable]
**Chronic diseases history**	[categorical variable]<br>1= 'yes, I have chronic diseases', 2= 'no, I don't have chronic diseases', 3= 'I am not sure'
**Self-reported illnesses over the previous two weeks**	[categorical variable]<br>1= 'yes, I have', 2= 'no, I don't have'
**3) Experience evaluation of health utilization**	
**Satisfaction for health service**	
**General feelings for health service**	[categorical variable]<br>0=I don't use it, 1=very bad feelings, 2=bad feelings, 3= neutral, 4=good feelings, 5=very good feelings
**Attitude of health staff**	[categorical variable]<br>0=I don't use it, 1=very bad feelings, 2=bad feelings, 3= neutral, 4=good feelings, 5=very good feelings
**Convenience and accessible for service**	[categorical variable]<br>0=I don't use it, 1=very bad feelings, 2=bad feelings, 3= neutral, 4=good feelings, 5=very good feelings

**Cost and affordable for service**	[categorical variable]<br>0=I don't use it, 1=very bad feelings, 2=bad feelings, 3= neutral, 4=good feelings, 5=very good feelings
**Health technology and skill level**	[categorical variable]<br>0=I don't use it, 1=very bad feelings, 2=bad feelings, 3= neutral, 4=good feelings, 5=very good feelings
**Self-reported changes in health service before and after settle-down (migrants only)**	
**general feelings**	[categorical variable]<br>0= I don't use it, 1= much worse, 2=worse, 3=no change, 4= better, 5=much better
**medical staff attitude**	[categorical variable]<br>0= I don't use it, 1= much worse, 2=worse, 3=no change, 4= better, 5=much better
**accessible**	[categorical variable]<br>0= I don't use it, 1= much worse, 2=worse, 3=no change, 4= better, 5=much better
**affordable and disease burden**	[categorical variable]<br>0= I don't use it, 1= much worse, 2=worse, 3=no change, 4= better, 5=much better
**health technology**	[categorical variable]<br>0= I don't use it, 1= much worse, 2=worse, 3=no change, 4= better, 5=much better
**4) Healthcare services utilization evaluation**	
**Basic Health insurance (BHI)**	[categorical variable]<br>1=EBMI, 2=RBMI, 3= no basic insurance
**Commercial insurance**	[categorical variable]<br>1= 'yes, I have', 2= 'no, I don't have'
**Health expenditure**	[continuous variable]
**Distance to nearest health institutions**	[categorical variable]<br>1=distance less than 1.0 km, 2= less than 2.0 km, 3= less than 3.0 km, 4= less than 4.0 km,<br>5= less than 5.0 km, 6= over 5.0km
**Traffic time to nearest health institutions**	[categorical variable]<br>0=no visit, 1= less than 15 min, 2= less than 20 min, 3=less than 30 min, 4= over 30 min
**Two-Weeks's clinic visit**	[categorical variable]<br>1= 'yes, I have', 2= 'no, I don't have'
**Self-medication behavior**	[categorical variable]<br>1= 'yes, I have', 2= 'no, I don't have'
**Hospitalization utility in last year**	[categorical variable]<br>1= 'yes, I have', 2= 'no, I don't have'

Appendix 5 The analysis results for propensity scores matching (PSM)

Appendix 5-1 Case Control Matching Statistics details

Match Type	Count
Exact Matches	0
Fuzzy Matches	290
Unmatched Including Missing Keys	239
Unmatched with Valid Keys	239
Sampling	without replacement
Log file	none
Maximize Matching Performance	yes

Appendix 5-2 Case Control Match Tolerances

Match Variables	Value	Fuzzy Match Tries	Incremental Rejection Percentage
Exact (All Variables)	0.000	19166.000	100.000
PS	0.020	19166.000	98.487

Note: Tries is the number of match comparisons before drawing.

Rejection percentage shows the match rejection rate. Rejections are attributed to the first variable in the BY list that causes rejection.

Appendix 6: differences analysis for regression coefficients (study croup vs. control group)

The differences analysis for regression coefficients (study croup vs. control group) was test in SPSSAU (Version 21.0) [Online Application Software] [R1].

test for equality estimation of regression coefficients [R2]

Variable	Type of group 0=control group, 1=study group		coefficients 1	coefficients2	Difference	t	p
age	0	1	0.02	0.026	-0.006	-0.808	0.42
Family members	0	1	0.212	0.227	-0.015	-0.197	0.844
Family income	0	1	-0.031	0.214	-0.245	-2.029	0.043*
Living expenditure	0	1	0.612	0.426	0.186	1.955	0.051
[gender=1]	0	1	-0.164	0.145	-0.309	-1.775	0.076
[marital status=1]	0	1	0.323	0.483	-0.16	-0.821	0.412
[BHIS=1]	0	1	0.896	0.059	0.838	4.279	0.000**
[BHIS=2]	0	1	1.097	-0.028	1.125	5.565	0.000**
[employee=1]	0	1	-0.419	0.106	-0.525	-2.024	0.044*
[employee=2]	0	1	-0.137	-0.318	0.181	0.54	0.589
[education=1]	0	1	0.207	-0.089	0.296	1.347	0.179
[education=2]	0	1	0.301	-0.266	0.567	3.255	0.001**
[Health status= 1]	0	1	0.515	0.285	0.229	1.136	0.257
[Health status=2]	0	1	0.467	0.37	0.097	0.562	0.575
[commercial insurance=1]	0	1	0.244	0.065	0.179	0.942	0.347

* p<0.05 ** p<0.01

Results for Chow Test [R3]

SSE			N			k	F	df 1	df 2	p
Total	0	1	Total	0	1					
428.984	163.25	232.433	464	216	248	16	2.272	16	432	0.003

Note:

R1 The SPSSAU project (2021). SPSSAU. (Version 21.0) [Online Application Software]. Retrieved from https://www.spssau.com.

R2 Cohen, J., & Cohen, P. (1983). Applied Multiple Regression/Correlation Analysis for the Behavioral Sciences (2nd ed.). Hillsdale, NJ: Lawrence Erlbaum

R3 Chow, G. C. (1960). Tests of equality between sets of coefficients in two linear regression. Econometrica.1960,28(3), 591-605.

Appendix 7 The related data for the 6th national health service statistic survey report

	**Urban-level in Central China**	**Rural-level in Central China**
**Sampling size**	40,099 people	41492
**Family size**	2.6 people	2.7
**Self-report health status**	78.7	74.4
**Self-report sick/illness in two-weeks [n (%)]**	12770 (31.8%)	13157(31.7%)
**Chronic diseases history [n (%)]**	11849(34.7%)	12572(37.5%)
**Basic health insurance coverage rate**	95.3%	97.5%
**Distance to nearest health institutions**		
-1.0 km	65.5%	56.4
-2.0 km	20.0%	21.8
-3.0 km	8.7%	12.6
-4.0 km	3.2%	4.3
-5.0 km	1.1%	1.3
5.0km-	1.5%	3.6
**Traffic time to nearest health institutions**		
-15 min	91.6%	88.1
-20min	5.0%	5.3
-30 min	2.9%	4.6
30min-	0.5%	2.0
**Two-Weeks's clinic visit**	19.2%	23.5%
**Hospitalization utility in last year**	12.9%	16.5%

Appendix 8 The annual per time cost for medical service in year 2018 at national level (RMB)

	**Clinic service**	**Stayin service**
**Hospitals**	274.1	9291.9
**Community health institutions**	132.3	3194.0
